# TIME-RESTRICTED EATING IN MASLD: THE IMPERATIVE TO PRESERVE MUSCLE MASS AND PREVENT WEIGHT CYCLING FOR SUSTAINABLE CLINICAL BENEFIT

**DOI:** 10.1590/S0004-2803.24612025-111

**Published:** 2026-05-22

**Authors:** Yusuf Bünyamin KETENCİ, Hakan DEMİRÖZ, Mehmet AKCA

**Affiliations:** * Department of Gastroenterology, Ondokuz Mayıs University, Samsun, Turkey.

**Keywords:** MASLD, time-restricted eating, sarcopenia, weight cycling, lifestyle intervention, liver fibrosis, MASLD, alimentação com restrição de tempo, sarcopenia, ciclo de peso, intervenção no estilo de vida, fibrose hepática

## Abstract

**Background and Objective:**

Time-restricted eating (TRE) and calorie restriction (CR) are effective lifestyle interventions for metabolic dysfunction-associated steatotic liver disease (MASLD). However, sarcopenia and weight cycling remain underappreciated threats that may offset their long-term benefits. This short communication reviews current evidence and clinical implications of these challenges.

**Methods:**

Sarcopenia, prevalent in MASLD, aggravates insulin resistance, systemic inflammation, and fibrosis progression. Recent randomized controlled trials, including Oh et al., reported significant muscle mass loss during TRE and CR interventions. Additionally, weight regain, affecting up to 60% of patients post-intervention, induces metabolic stress, promotes hepatic fat re-accumulation, and may accelerate disease progression.

**Conclusion:**

MASLD interventions should integrate muscle-preserving strategies-resistance exercise, adequate protein intake-and long-term weight maintenance support. Future trials must measure functional muscle outcomes, assess fibrosis progression, and evaluate sustainable behavioral strategies to prevent weight cycling.

## HIGHLIGHTS

• TRE and CR yield comparable reductions in hepatic fat, body weight, and visceral adiposity in MASLD.

• Both strategies can lead to skeletal muscle loss, increasing the risk of sarcopenia.

• Weight regain after interventions is common and may aggravate steatosis and fibrosis progression.

• Sustainable MASLD care requires resistance exercise, adequate protein intake, and monitoring of muscle mass and function.

## RESUMO

**Contexto e Objetivo:**

A alimentação com restrição de tempo (TRE) e a restrição calórica (CR) são intervenções eficazes de estilo de vida para a doença hepática gordurosa associada à disfunção metabólica (MASLD). No entanto, a sarcopenia e o ciclo de peso permanecem ameaças subestimadas que podem anular seus benefícios a longo prazo. Esta comunicação breve revisa as evidências atuais e as implicações clínicas desses desafios.

**Métodos:**

A sarcopenia, prevalente na MASLD, agrava a resistência à insulina, a inflamação sistêmica e a progressão da fibrose. Ensaios clínicos randomizados recentes, incluindo Oh et al., relataram perda significativa de massa muscular durante intervenções com TRE e CR. Além disso, o reganho de peso, que afeta até 60% dos pacientes após a intervenção, induz estresse metabólico, promove o reacúmulo de gordura hepática e pode acelerar a progressão da doença.

**Conclusão:**

As intervenções para MASLD devem integrar estratégias de preservação muscular - exercício de resistência, ingestão adequada de proteína - e suporte para manutenção de peso a longo prazo. Ensaios futuros devem medir desfechos funcionais musculares, avaliar a progressão da fibrose e investigar estratégias comportamentais sustentáveis para prevenir o ciclo de peso.

## INTRODUCTION

Metabolic dysfunction-associated steatotic liver disease (MASLD)-previously nonalcoholic fatty liver disease-has become the most prevalent chronic liver disease globally. It is strongly linked to obesity, type 2 diabetes mellitus, dyslipidemia, and other elements of metabolic syndrome^1^. Because of that, lifestyle interventions represent the cornerstone of MASLD management.

Calorie restriction (CR) has long been advocated to achieve weight loss and metabolic improvement. More recently, time-restricted eating (TRE), which limits calorie intake to a specific daily window without necessarily altering caloric content, has emerged as a popular and potentially effective dietary approach^2^. The rationale for TRE stems from evidence that aligning food intake with circadian rhythms can improve metabolic regulation. Importantly, CR should be implemented gradually to avoid rapid weight loss, which can negatively affect hepatic metabolism. The macronutrient composition of CR should be tailored according to individual patient status, body composition, and activity level. Notably, myosteatosis is a frequent comorbidity in MASLD, highlighting the need to preserve muscle quality and function during dietary interventions.

In their recent randomized controlled trial, Oh et al.^3^ demonstrated that both TRE and CR produced comparable reductions in hepatic fat content, body weight, and visceral adiposity in patients with MASLD over a 16-week period compared to standard of care. These findings reinforce TRE’s potential as a feasible and effective tool in MASLD therapy.

However, two important challenges have emerged from the study and other recent literature: firstly, significant loss of skeletal muscle mass during both TRE and CR interventions; secondly, high rates of weight regain (weight cycling) after intervention cessation. Both phenomena have profound clinical implications in MASLD, threatening to negate initial benefits and potentially accelerate disease progression.

### Sarcopenia and MASLD

Sarcopenia-loss of skeletal muscle mass and function-is increasingly recognized as a major determinant of outcomes in chronic liver disease^4^. In MASLD, sarcopenia prevalence is estimated between 15-25%, with higher rates in elderly populations and those with coexisting metabolic comorbidities^5^. Mechanistically, sarcopenia can exacerbate insulin resistance. Reduced muscle mass diminishes glucose uptake, impairing glycemic control and favoring hepatic lipid accumulation. Pro-inflammatory cytokines from adipose tissue, combined with reduced myokine production, create a pro-fibrotic milieu. Several longitudinal studies demonstrate sarcopenia as a predictor of advanced fibrosis and adverse hepatic events^4^.

In the Oh et al. trial^3^, muscle mass decreased by approximately 2.1% with TRE and 1.8% with CR, compared to only 0.6% with standard care. While modest in absolute terms, such reductions over a short period are clinically relevant, especially if compounded by repeated weight loss cycles. Notably, most dietary intervention studies, including TRE trials, assess lean body mass but omit functional endpoints such as grip strength or chair-stand time-measures essential for diagnosing and monitoring sarcopenia^4^. TRE may be particularly suitable for patients with favorable psychological and behavioral profiles, as it can facilitate improved control of food intake and positively influence circadian rhythms.

### Weight cycling and MASLD

Weight cycling, or repeated loss and regain of body weight, is common after structured lifestyle interventions. Oh et al.^3^ reported that ~60% of intervention participants regained a substantial portion of lost weight within six months.

Clinical and experimental evidence suggests weight cycling can induce metabolic stress. Re-weight gain can reduce basal metabolic rate and favor fat over lean mass re-accumulation^6^. Increased de novo lipogenesis and oxidative stress during regain may worsen steatosis more than baseline levels^7^. Fluctuating metabolic states may increase stellate cell activation and extracellular matrix deposition^8^. A large Korean cohort study found that individuals with significant weight variability had higher risks of incident MASLD and fibrosis progression, independent of baseline body mass index^9^.

### Clinical and research implications

Given the dual threats of sarcopenia and weight cycling, MASLD interventions should not focus solely on calorie restriction or meal timing. A multidisciplinary, long-term approach is needed. Combining resistance and aerobic modalities, ensuring protein adequacy and nutrient density, regular monitoring of body composition and muscle function, and behavioral and nutritional follow-up to sustain weight loss must be incorporated into MASLD lifestyle interventions.

Future TRE studies should include functional muscle assessments, body composition changes beyond fat mass, evaluate fibrosis progression with non-invasive and/or histologic endpoints, and assess long-term adherence and relapse risk. Another important consideration is the impact of ethnicity on MASLD development and progression. For example, individuals of Asian descent may develop steatohepatitis at lower levels of overweight compared to Hispanic populations, emphasizing the need for culturally tailored dietary strategies.

## CONCLUSION

TRE is an effective dietary strategy in MASLD, yielding comparable benefits to CR. Yet, skeletal muscle loss and frequent weight regain may undermine its long-term value. Integrating muscle-preserving exercise, adequate protein intake, and sustained behavioral support is essential to maximize benefit and minimize harm. Future research should clarify the long-term hepatic and metabolic impacts of TRE while proactively addressing these risks.

## Data Availability

Not applicable - The study did not use research data
